# Report of a Case of Vesico Vaginal Fistula

**Published:** 1859-06

**Authors:** Robert Battey

**Affiliations:** Rome, Georgia


### ARTICLE III.

TRANSACTIONS OF THE MEDICAL ASSOCITIAON OF GEORGIA.

*Report of a Case of Vesico Vaginal Fistula.* Presented to the Medical Association of Georgia, by ROBERT BATTEY, M. D., of Rome, Georgia.

Caroline, a negro, aged 25, mother of two children, the first living to the age of six months, the second was delivered by use of the perforator three years since, from which time she has had a regular stillicidium of urine. For three weeks prior to May 7th, 1858, she had observed what she supposed to be the head of a bone, projecting down into the vagina, and gradually making its way outwards between the labia. When removed, the foreign body proved to be a vesical calculus of the variety denominated "fusible," rather club shaped in its form, and crystaline in structure, weighing 229 grains.

The perineum was greatly thickened and indurated from the contact of the stone; uterus prolapsed, and cervix ulcerated; fistulous opening into the bladder extending from the median line laterally, towards the ramus of the right ischium, readily admits the first joint of the index finger. Prescription—quinia and mineral acid, with the use of belladonna ointment to perineum.

June 22d.—Bowels having been moved out freely with castor oil on yesterday, patient was examined and submitted to operation, after the manner of Sims, as modified by Bozeman—using the leaden button, with three points of silver suture—patient put to bed, and two grains opium administered.

Desiring to render the patient as comfortable as possible, and protect the bedding from the urinary secretion, a bottle was prepared with two glass tubes, having one end of each drawn out to a capillary opening, passed down through the cork, and connected by elastic tubing, to the orifice of Sims' catheter. This apparatus received the urine perfectly as it passed from the catheter, and accomplished the objects in view, most satisfactorily; but, as the subsequent history proved, at the expense of much unnecessary suffering and risk to the patient.

23d.—Rested well last night; feels quite comfortable; took cold during the night, and is coughing occasionally; catheter and bottle works admirably; opium continued.

25th.—Doing well, except the cough, which is becoming quite troublesome; a little dribbling of urine through vagina yesterday, none since; prescribed paliative for cough.

26th.—Catamenia made its appearance last night (she has been quite irregular for some time); cough continues troublesome; pulse normal; tongue furred; complains a little of tenderness in the hypogastrium; examined sutures and find them apparently in good condition, with little or no urine passing fistula.

27th.—Doing well; no urine passing fistula; catamenia continues; pulse 80; tongue furred; vitiated taste; cough troublesome; urine turbid and alkaline; prescribed elixir vitriol, cough mixture, and opium continued.

28th.—Headache; urine scanty; sutures examined, centre one cut out. Deeming the operation a failure, the sutures were removed, and fistula found in better condition than expected—union is perfect at the sides, but rather defective in the centre. Constipation maintained and treatment continued.

30th.—Fever; abdominal tenderness increasing; prescribed enema, blue pill, poultice to abdomen.

July 1st.—Considerable fever; cough subsiding; passed more than an ounce of blood through the catheter this morning; prescribed neutral mixture, discontinued catheter.

2d.—Pulse normal; urine sanguinolent; retains it perfectly; passed it voluntarily several times during the night.

3d.—Pulse normal; pain in right ovary; tenderness in right hypochodrium; urine sanguinolent; retained perfectly; catamenia continues; two or three dysenteric discharges, with painful tenesmus through the day; prescribed blue pill and dovers powder.

5th.—Doing well; hæmaturia continues; was allowed to leave her bed on yesterday.

28th.—Examined cicatrix, firm and smooth, save in one spot, a small fossa, from the centre of which, a little urine dribbles when the bladder is compressed; opening is only perceptible by causing urine to ooze through it. She retains her urine perfectly at night, and when sitting down; but it dribbles when upon her feet. She voided six fluid ounces to-day, just prior to inspection, having been upon her feet for some hours.

It will be observed that the sutures were removed upon the 6th day after operation, constipation upon the 8th and catheter upon the 9th. Her comparatively comfortable condition has caused her to be neglected for some time. It is now proposed by refreshing the sides of the little fossa alluded to, and inserting a single stitch with a small button to complete the cure.

